# Validity and Reliability of Stillbirth Data Using Linked Self-Reported and Administrative Datasets

**DOI:** 10.2188/jea.JE20140032

**Published:** 2015-01-05

**Authors:** Alexis J. Hure, Catherine L. Chojenta, Jennifer R. Powers, Julie E. Byles, Deborah Loxton

**Affiliations:** Research Centre for Gender Health and Ageing, School of Medicine and Public Health, University of Newcastle, Australia

**Keywords:** data, kappa, linkage, reliability, self-report, sensitivity, specificity, stillbirth, validity

## Abstract

**Background:**

A high rate of stillbirth was previously observed in the Australian Longitudinal Study of Women’s Health (ALSWH). Our primary objective was to test the validity and reliability of self-reported stillbirth data linked to state-based administrative datasets.

**Methods:**

Self-reported data, collected as part of the ALSWH cohort born in 1973–1978, were linked to three administrative datasets for women in New South Wales, Australia (*n* = 4374): the Midwives Data Collection; Admitted Patient Data Collection; and Perinatal Death Review Database. Linkages were obtained from the Centre for Health Record Linkage for the period 1996–2009. True cases of stillbirth were defined by being consistently recorded in two or more independent data sources. Sensitivity, specificity, positive predictive value, negative predictive value, percent agreement, and kappa statistics were calculated for each dataset.

**Results:**

Forty-nine women reported 53 stillbirths. No dataset was 100% accurate. The administrative datasets performed better than self-reported data, with high accuracy and agreement. Self-reported data showed high sensitivity (100%) but low specificity (30%), meaning women who had a stillbirth always reported it, but there was also over-reporting of stillbirths. About half of the misreported cases in the ALSWH were able to be removed by identifying inconsistencies in longitudinal data.

**Conclusions:**

Data linkage provides great opportunity to assess the validity and reliability of self-reported study data. Conversely, self-reported study data can help to resolve inconsistencies in administrative datasets. Quantifying the strengths and limitations of both self-reported and administrative data can improve epidemiological research, especially by guiding methods and interpretation of findings.

## INTRODUCTION

Validity is defined as the property of being true, correct, and conforming with reality. Reliability is concerned with the consistency, rather than the accuracy, of a measure. Assessing both the accuracy (validity) and agreement (reliability) of self-reported data is essential in conducting good-quality epidemiological research. However, assessing validity generally relies on the availability of a ‘gold standard’ for comparison.

Patients or study participants are often asked to self-report their reproductive history, whether for clinical or research purposes. It is important to know the validity and reliability of these reports. One study of 754 women in the United States assessed the reliability of self-reported reproductive data against medical records.^1^ In this study, Olsen et al showed very high agreement between self-reported data and medical records for the number of live births (kappa 1.0), number of previous pregnancies (kappa 0.9), gestational age at birth (correlation 0.8), and number of miscarriages (kappa 0.7). However, the reliability of self-report of stillbirths has not previously been assessed. Furthermore, while medical records serve as an important source of information, they do not serve as a gold standard alone.^1^ Therefore, the validity of reproductive data using a combination of sources should also be assessed.

### What is stillbirth?

Australia defines stillbirth as the death of a fetus prior to birth, where the fetus is 20 or more completed weeks gestation or 400 grams or more in birth weight.^2^ Fetal death is indicated because the fetus does not breathe or show any other signs of life, such as a pulse, pulsation of the umbilical cord, or definite movement of voluntary muscles.^2^ There are legal implications for reporting fetal deaths, and this definition of stillbirth is used as an eligibility criterion for receiving certain government entitlements in Australia.^3^

### Project aim

We have previously published research showing that the rate of self-reported stillbirth in the Australian Longitudinal Study of Women’s Health (ALSWH) cohort, a study including women born in 1973–1978, was higher than national perinatal statistics: 11.0^4^ versus 7.8^5^ stillbirths per 1000 live births, respectively. The reason for this unusually high rate of self-reported stillbirth may be the result of measurement error, and further investigation is required if the ALSWH data are to be used appropriately in future studies. However, because there is no gold standard for measuring stillbirths, the comparator also requires evaluation. Therefore, the current study aimed to test the validity and reliability of self-reported stillbirth data linked with three state-based administrative datasets. A secondary aim was to test the validity and reliability of each administrative dataset, comparing it to consensus stillbirth data drawn from multiple sources.

## METHODS

### New South Wales (NSW) administrative datasets

Although there is overlap in the content recorded in each of the following administrative datasets, they are considered to be independent of one another. There are different recording systems in place for each dataset, and the records are entered by different staff within the health system. While the data sources are technically independent of one another, they are also correlated, as are the administrative and self-report data.

#### Midwives Data Collection (MDC)

In NSW, perinatal deaths are recorded in the NSW Perinatal Data Collection, previously known as the NSW MDC until 31 December, 2010.^6^ The MDC was developed in 1986 and provides information about pregnancy care, services, and outcomes.^7^ Data collection covers all births of at least 400 grams birth weight or at least 20 weeks gestation.^8^ A separate record is completed for each baby in the case of a multiple birth. The information is recorded by either the midwife or medical practitioner.^7^ It includes demographic, medical, and obstetric information on the mother and information on the labour, delivery, and condition of the offspring. The MDC does not receive notifications when a mother from NSW gives birth in another state.^8^

#### Admitted Patient Data Collection (APDC)

Stillbirths are also coded in the NSW APDC from medical records by clinical coders upon a patient’s discharge from hospital. The APDC includes records for all hospital separations, meaning discharges, transfers, and deaths.^9^ Separations are recorded from all public and private hospitals, public multi-purpose services, and private day-procedure centres.^9^ The APDC records include a range of demographic data, admission and separation dates, and International Classification of Diseases (ICD-10) codes^10^ on reasons for the admission, significant co-morbidities or complications, and procedures performed during the admission. The ICD-10 codes are used to describe the number of stillbirths for multiple births (eg Z37.3 signifies twins, one liveborn and one stillborn), if needed. The APDC includes data for NSW residents hospitalised interstate; however, names and addresses are not included on these records and therefore cannot be included in record linkage studies.^9^

#### Perinatal Death Review Database (PDRD)

When a baby is stillborn or dies within 28 days of birth, a notification that is separate to the perinatal data collection is made to the PDRD by the attending midwife or medical practitioner. Perinatal deaths are independently and confidentially reviewed by a subgroup of the NSW Ministerial Maternal and Perinatal Committee, and confirmed cases are recorded in the PDRD.^9^ Stillbirths and neonatal deaths are classified according to the Perinatal Society of Australia and New Zealand’s Perinatal Mortality Classifications.^11^ From 2000 through 2005, only perinatal deaths of at least 500 grams birth weight or 22 weeks gestation were reviewed, despite the lower cut-points (ie >400 grams or >20 weeks gestation) defining stillbirth in the MDC and APDC. It is important to note that the definition of stillbirth as a medical term was consistent for NSW across the entire timeframe of our study. The purpose of the slightly higher threshold in the PDRD was to focus attention on deaths that were more likely to be preventable.^12^ From 2006, the PDRD included all stillbirths.^9^

### Self-reported study data

#### The Australian Longitudinal Study of Women’s Health (ALSWH): cohort born in 1973–1978

The ALSWH is a government-funded initiative established to examine demographic, social, physical, psychological, and behavioural variables and their effect on women’s health, well-being, and use of health services.^13^ Full details of the ALSWH’s prospective study design and recruitment have been reported elsewhere.^13^^–^^15^ Briefly, the ALSWH recruited 14 247 women aged 18 to 23 years (the cohort born in 1973–1978) at the baseline survey in 1996. Potential participants were randomly selected from the national health insurance (Medicare) database, except that women from non-urban areas were intentionally over-sampled.^13^ An invitation to participate was mailed out, and those who consented were deemed broadly representative of women of the same age within the Australian population.^15^

ALSWH surveys were mailed to the cohort born in 1973–1978 in 1996 (Survey 1), 2000 (Survey 2), 2003 (Survey 3), 2006 (Survey 4), and 2009 (Survey 5). Pregnancy, birth, and child data were collected at each of the five surveys. Survey 5 was mailed out in March 2009, and responses were returned by 8,200 women over the following 15 months (to May 2010); however, 90% of surveys were returned by December 2009. The response rate to Survey 5 was 58% of those who completed Survey 1.^4^ Compared to non-responders, more women who completed Survey 5 had never smoked (54% versus 45%) and had at least 12 years of education (70% versus 65%) at baseline.^4^ However, women who completed Survey 5 were not meaningfully different from non-responders in terms of age, marital status, or area of residence at baseline.^4^

#### Stillbirths in the ALSWH

The stillbirths reported in the ALSWH were compared against the administrative records in the MDC, APDC, and PDRD. Women were asked to recall their history of stillbirth in all ALSWH surveys from 2000 (Survey 2) onwards. The question was worded ‘How many times have you had each of the following?’ with ‘Stillbirth’ listed after ‘Live birth’. Response categories were ordinal and ranged from 0 up to ‘5 or more’. Dates of birth were also provided for all previous births in 2003 (Survey 3), 2006 (Survey 4), and 2009 (Survey 5), with duplicate entries requested for multiple births. ALSWH women who had ever reported one or more stillbirths in any dataset and had completed one or more survey(s) while living in NSW were included in our validity and reliability analyses. Consistency of the self-reported data was assessed by referring to the responses in each participant’s ALSWH survey(s) in chronological order.

### Data linkage: the Centre for Health Record Linkage (CHeReL)

The CHeReL was established in 2006 and is jointly managed by the Cancer Institute NSW and the NSW Ministry of Health.^16^ The CHeReL brings together information from two or more sources, including longitudinal studies like the ALSWH. Custodians of each nominated dataset for linkage provide the CHeReL with an encrypted source record number and demographic details for each person.^16^ Clinical data are not provided to the CHeReL; they remain with the data custodians. The CHeReL links records using probabilistic matching of the demographic details.^16^ Participant privacy and confidentiality are maintained throughout data linkage because: (i) the CHeReL staff performing the linkage using demographic variables but do not have access to the clinical information about individuals; (ii) data custodians only have access to data within their data collections; and (iii) researchers receive data which contains no identifying variables or variables that provide a link back to the CHeReL’s Master Linkage Key.^16^ The Master Linkage Key is the CHeReL’s system for continuously updating links within and between core health-related datasets in NSW.

### Linkage of the ALSWH and NSW administrative datasets

Record linkage was performed in May 2011 by the data custodians of each dataset. The self-reported and administrative datasets included in these analyses are listed in Table 1. Only five years of data were available for the PDRD, whereas 14 years of prospective data were available from the ALSWH. Validity and reliability measures include only the records that were matchable according to the date limits outlined in Table 1 and Figure. For example, only stillbirths that occurred between July 2000 and December 2004 were used to compare the PDRD against the three other datasets. In total, there was four and a half years of overlap for all four datasets, four and a half years of overlap for three datasets, four and a half years of overlap for two datasets, and only one year of ALSWH data with no overlap (Figure).

**Figure.**
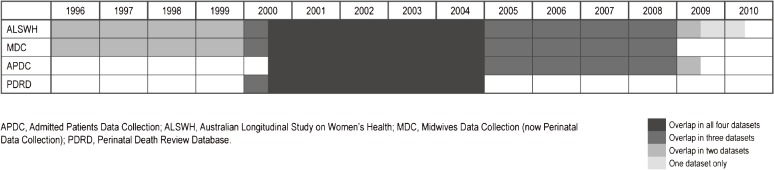
Timelines for the linked self-reported and administrative datasets on stillbirths in New South Wales, Australia

**Table 1.** Stillbirths in New South Wales, Australia: timelines and cases for linked self-reported and administrative datasets

Datasets	Data from	To	Number of Stillbirths
***Self-reported data***			
Australian Longitudinal Study on Women’s Health, *New South Wales only*^a^ (*n* = 4374 women)	1 July 1996	31 May 2010	45^b^
***State-based administrative data***			
Midwives Data Collection (*n* = 2166 women)	1 January 1996	31 December 2008	22
Admitted Patient Data Collection (*n* = 2003 women)	1 July 2000	30 June 2009	18
Perinatal Death Review Database^c^ (*n* = 10 women)	1 January 2000	31 December 2004	10
TOTAL			53^d^

^a^Includes all participants who answered ≥1 Australian Longitudinal Study on Women’s Health survey while living in New South Wales and/or had ≥1 administrative record.

^b^*n* = 3 women self-reported more than 1 stillbirth (*n* = 41 women).

^c^Only reviewed cases ≥22 weeks gestation or 500 g birth weight until January 2006.

^d^*n* = 49 women in total.

### Ethics and consent

Ethics approvals for the full ALSWH were obtained from the Human Research Ethics Committees of the Universities of Newcastle and Queensland, and written informed consent was provided by participants. Ethics approval for data linkage of the NSW administrative and ALSWH datasets was received from the NSW Population and Health Services Research Ethics Committee, and approval was registered with the University of Newcastle. Consent for data linkage was provided on an opt-out basis. That is, all women who provided written informed consent to participate in the 1973–1978 cohort of the ALSWH were included in data linkage, unless they explicitly responded that they did not want their records to be linked.

### Determining validity

Validity can be determined for cases of stillbirth because there is objective evidence, generally from a hospital admission. However, data errors may be introduced during record entry or data linkage. Because there is no gold standard reference for true cases of stillbirth, we used the combined power of the four linked data sources (three administrative datasets and one self-report). We applied the criterion that two of two, two of three, or three of four independent data sources must agree before concluding a confirmed stillbirth. In this way each dataset could be assessed against a more objective measure of stillbirth than any one dataset could provide.

The MDC, which records both gestational age at birth and birth weight, was used to look for missing cases of stillbirth (false negatives) in the PDRD. If a stillbirth of at least either 22 weeks gestation or 500 grams birth weight was recorded in the MDC but missing from the PDRD, the APDC and ALSWH were used to classify the birth as either a confirmed stillbirth (ie false negative in the PDRD) or false positive in the MDC, if at least one additional dataset was available.

### Statistical analysis

Data merges and analyses were performed using Intercooled Stata, version 11 (StataCorp, College Station, TX, USA). The validity and reliability analyses used the number of women as the denominator, rather than the number of stillbirths, and were restricted to women with a stillbirth noted in at least one dataset.

Validity for confirmed cases was assessed by calculating sensitivity, specificity, positive predictive value (PPV), and negative predictive value (NPV) for each dataset. Sensitivity is defined as the proportion of women who had one or more confirmed stillbirths and were correctly classified by the dataset. Specificity is defined as the proportion of women who did not have any stillbirths and were correctly classified by the dataset. The PPV is defined as the proportion of women who actually had a stillbirth (true positives) of all women reporting stillbirth (true plus false positives), and NPV is the proportion of women who actually did not have a stillbirth (true negatives) of all women not reporting a stillbirth (true plus false negatives).

### Determining reliability

Reliability between the self-reported and administrative datasets was determined using percentage agreement and kappa statistics. Reliability measures were provided for the confirmed cases of stillbirth, in addition to cases where the data for each pair of datasets overlapped. Each administrative dataset was compared to all other datasets. The kappa statistic measures the extent of the exact agreement, adjusting for chance agreement.^17^ Values greater than 0.75 represent excellent agreement, values of 0.75 to 0.40 represent moderate agreement, and values less than 0.4 represent poor agreement.^17^ Negative kappa statistics result when agreement occurs less often than predicted by chance alone.^18^

## RESULTS

Nine percent (*n* = 1186) of the entire ALSWH cohort declined data linkage. Linked data were available for 4374 ALSWH women from NSW. The samples from each dataset and the respective number of stillbirths reported are shown in Table 1. The self-reported dataset contained the greatest number of women (*n* = 41) and stillbirths (*n* = 45), double that of any administrative dataset.

### Validity of stillbirth data

The total number of women with 1 or more recorded stillbirths from any of the four datasets was 49, and a total of 53 stillbirths were recorded (Table 1). Of those, 24 women (49%) had their data confirmed in two or more independent datasets (for example, recorded in the MDC and self-reported in ALSWH). Ten (20%) were found not to have had stillbirths: four women in the ALSWH had confirmed cases of spontaneous miscarriage or medical termination (prior to 20 weeks gestation); three had inconsistently self-reported stillbirths in the ALSWH surveys, with no evidence of stillbirth in any administrative dataset; data for two women were miscoded in the APDC; and one linkage error occurred where data had merged for two different women under the same identifier in the MDC. Data for the remaining 15 (31%) women with reported stillbirths could neither be confirmed nor refuted, primarily because data were conflicting in two datasets and no third source was available (*n* = 6), or the stillbirth was reported in the ASLWH as having occurred prior to 1996 (*n* = 3, with no overlap in datasets). Other reasons stillbirth data could not be validated included ALSWH women living outside of NSW for one or more surveys with no later NSW-based data available to cross-check (*n* = 5), and a Master Linkage Key error within the CHeReL where all records were missing from the administrative datasets (*n* = 1).

Table 2 presents the sensitivity, specificity, PPV, and NPV for each dataset compared with the confirmed cases of stillbirth. No dataset was 100% accurate. The ALSWH had particularly high sensitivity but had low specificity, meaning that women who had a stillbirth always self-reported it, though some women who had not had a stillbirth also self-reported having experienced this. Overall, MDC performed the best, with both high sensitivity (96%) and specificity (90%). The accuracy of the MDC and/or APDC datasets were slightly inflated because discrepancies in two cases could not be resolved as either stillbirths (MDC) or miscarriages (APDC) using a third data source. These errors could not have been detected if either the MDC or APDC had been used as the reference instead of taking at minimum an agreement of two independent data sources.

**Table 2.** Confirmed^a^ cases of stillbirth: validity of self-reported and administrative datasets in New South Wales, Australia

	Confirmed stillbirth (*n*)		Not a stillbirth (*n*)		Positive Predictive Values % [95% CI]	
Recorded stillbirth (test positive)	ALSWH: 21	MDC: 22	ALSWH: 7	MDC: 1	ALSWH: 75 [59, 91]	MDC: 100
	APDC: 16	PDRD: 10	APDC: 2	PDRD: 0	APDC: 89 [76, 100]	PDRD: 100

					Negative Predictive Values % [95% CI]	

No recorded stillbirth (test negative)	ALSWH: 0	MDC: 1	ALSWH: 3	MDC: 9	ALSWH: 100	MDC: 90 [71, 100]
	APDC: 0	PDRD: 2	APDC: 8	PDRD: 10	APDC: 100	PDRD: 83 [62, 100]

	Sensitivity % [95% CI]		Specificity % [95% CI]			

	ALSWH: 100%	MDC: 96% [87, 100]	ALSWH: 30 [16, 58]	MDC: 90 [71, 100]		
	APDC: 100%	PDRD: 83 [62, 100]	APDC: 80 [55, 100]	PDRD: 100

APDC, Admitted Patients Data Collection; ALSWH, Australian Longitudinal Study on Women’s Health; CI, confidence interval; MDC, Midwives Data Collection (now called Perinatal Data Collection); PRDR, Perinatal Death Review Database.

^a^Two or more independent data sources must agree before concluding a confirmed stillbirth. Includes only women who self-reported or had a stillbirth recorded in one or more datasets. True positives and true are negatives are shaded in grey.

### Reliability of stillbirth data

Table 3 shows the percentage agreement and kappa statistics for each dataset compared against just the confirmed cases of stillbirth. The ALSWH was the least reliable, with 77% agreement between self-reported versus confirmed cases. The MDC had the highest percentage agreement between recorded and confirmed stillbirth cases, at 94%.

**Table 3.** Confirmed^a^ cases of stillbirth: reliability of self-reported and administrative datasets in New South Wales, Australia

	*n*^b^	% Agreement [95% CI]	Kappa [95% CI]
***Self-reported data***			
Australian Longitudinal Study on Women’s Health	31	77 [63, 92]	0.37 [0.05, 0.69]

***State-based administrative data***			
Midwives Data Collection	33	94 [86, 100]	0.86 [0.66, 1.00]
Admitted Patient Data Collection	26	92 [82, 100]	0.83 [0.62, 1.00]
Perinatal Death Review Database	22	91 [79, 100]	0.82 [0.59, 1.00]

CI, confidence interval.

^a^Two or more independent data sources must agree before concluding a confirmed stillbirth.

^b^Number of women rather than stillbirths.

Table 4 shows the percentage agreement and kappa statistics between each pair of datasets, not relying on confirmed cases. The reliability of the self-reported stillbirth data compared to the administrative datasets was poor. After ALSWH data cleaning, the agreement with administrative datasets increased from 55%–66% to 65%–75%, though the kappa statistics remained low (<0.4). The administrative datasets only performed moderately well themselves. Errors included data missing from the MDC and one stillbirth that should have been reviewed in the PDRD but was not. Smaller errors, like duplicating a pregnancy number and then skipping one for subsequent births, were also detected in the MDC.

**Table 4.** Reliability of self-reported and administrative datasets on stillbirths in New South Wales, Australia

	Australian Longitudinal Study on Women’s Health [95% CI]						Midwives Data Collection [95% CI]			Admitted Patient Data Collection [95% CI]		

	Raw			Cleaned^a^								

	*n*^b^	%Agreement	Kappa	*n*^b^	%Agreement	Kappa	*n*^b^	%Agreement	Kappa	*n*^b^	%Agreement	Kappa
Midwives Data Collection	32	66 [49, 82]	0.14 [−0.13, 0.41]	28	75 [59, 91]	0.25 [−0.12, 0.62]						

Admitted Patient Data Collection	26	58 [39, 77]	−0.03 [−0.31, 0.26]	22	68 [49, 88]	0.05 [−0.35, 0.45]	28	79 [63, 94]	0.53 [0.21, 0.86]			

Perinatal Death Review Database	20	55 [33, 77]	0.21 [−0.02, 0.45]	16	69 [46, 91]	0.38 [0.02, 0.73]	22	91 [79, 100]	0.82 [0.59, 1.00]	22	82 [66, 98]	0.65 [0.35, 0.94]

CI, confidence interval.

^a^Women reporting an inconsistent number of stillbirths over multiple surveys were removed from the analysis (*n* = 4).

^b^Number of women rather than stillbirths.

## DISCUSSION

We present, for the first time, a validity and reliability study of self-reported and state-recorded stillbirths. We have used data from a prospective longitudinal cohort, linked to three administrative datasets for NSW, Australia, because there is no gold standard for stillbirth data. Using our methods, almost 70% of cases were able to be classified as either a confirmed stillbirth or false negative. The results clearly demonstrate that no single source is entirely accurate and reliable; no single dataset can perform as the gold standard in isolation. However, the administrative datasets, in particular the MDC (now known as the Perinatal Data Collection), do perform better than the self-reported stillbirth data when balancing accuracy and precision, if only one dataset is available. The second striking finding in this study is that stillbirth was self-reported with 100% sensitivity. Beyond our focus on stillbirth, this work can serve as an example of methodology for data linkage studies, particularly those involving perinatal administrative data.

In the self-reported dataset (the ALSWH cohort), women who objectively had a stillbirth always reported it (ie, 100% sensitivity). This is an important and positive finding, because there is evidence that mothers who experience stillbirth face social stigma, blame, and marginalisation, which lead to underreporting.^19^ The cultural practice in Australia is such that parents and family members may hold, dress, and name their stillborn baby, prior to commemoration with a funeral. They will also receive a birth certificate. These cultural practices, in addition to an evidence-based medical system, may contribute to women feeling comfortable recording their stillbirth.

The accuracy of the stillbirth data was reduced by some women who had not had a stillbirth recording that they had, or by misreporting a late spontaneous miscarriage or medical termination as a stillbirth (ie, low specificity). The administrative (hospital) datasets also echoed some confusion in the classification of stillbirth versus late spontaneous miscarriage or medical termination, which suggests that health professionals also experience difficulty in classifying borderline cases. In addition to the emotional ramifications, the Australian Government has paid a maternity allowance for all live- and stillbirths since February 1996,^3^ whereas miscarriages or terminations before 20 weeks gestation do not result in any payment. Furthermore, stillbirths are eligible for a birth certificate, whereas fetuses lost before 20 weeks gestation are not.^20^ Women experiencing a late miscarriage want greater recognition of their loss,^20^ even calling for the definition of stillbirth to be revised to an earlier gestational age. This may help to explain why some report miscarriage as a stillbirth. Our findings are not only relevant to the ALSWH but are important for clinicians who work in reproductive health, especially those dealing directly with women experiencing miscarriage and stillbirth.

Data for four of the seven women who misreported stillbirths in the ALSWH were able to easily be identified and removed through thorough data cleaning protocols. In particular, inconsistency in the number of stillbirths reported over time was used to identify misreporting. In longitudinal studies, the complete survey history should be considered during data cleaning. When this is not available, considering a variety of related questions within the same survey can also help in identifying erroneous data. In the case of the ALSWH, researchers who use the self-reported stillbirth data without linked administrative records should perform thorough data cleaning to exclude cases of over-reporting, or consider combining stillbirth and miscarriage into a single ‘fetal loss’ variable, depending on relevance to the research question.

Stillbirths are a relatively rare but devastating event. Flenady et al recently estimated that 1 in every 200 women in high-income countries who reach 22 weeks gestation will have a stillborn baby.^21^ We have drawn on a variety of large datasets containing more than 2000 women with linked data to obtain enough stillbirth cases for assessing both validity and reliability of each source. Nine years of overlap were available for three or more datasets; however, data could neither be confirmed nor refuted for 30% of the women in our sample. It is possible that the cases of confirmed stillbirth might be more accurate during the period where the four datasets overlapped compared to just two. However, we suggest there are rapidly diminishing returns on using more than two datasets. In fact, there were no cases where four datasets were available, with a stillbirth recorded in two and absent in the other two. Hence, the cases would have been equally classifiable with only three datasets.

Our final sample (*n* = 49 women) is small, which may result in imprecise validity and reliability estimates. Data linkage is the best way to obtain an adequate sample to test for predictors of stillbirth.^22^ However, the present study provides a useful evaluation of the administrative datasets used in linkage and some insights into identifying and removing errors. While the specific results are not generalizable beyond the datasets contained within this paper, our study demonstrates a clear need for validation of self-reported research data, in addition to ongoing monitoring and evaluation of the information documented within state-based or national healthcare records. Quantifying the strengths and limitations of any dataset, be it self-reported or administrative, will improve the quality of epidemiological research, especially by guiding appropriate methodology and interpretation of the findings.

Self-reported data have the advantage of being readily available and may provide much more information than any single administrative dataset alone. We have shown some trade-off in accuracy of the data against the amount of data available for analysis. In the present analysis, we were only able to link reproductive data for women in NSW. However, NSW is Australia’s most populous state, comprising just over one-third of the national population. Efforts are underway to link other areas of the ALSWH data with each of the states’ and territories’ data, making the data linkage more nationally representative and facilitating further validity and reliability studies.

### Conclusions

Data linkage can and should be used not only to test the validity and reliability of self-reported data from epidemiological studies but also to cross-check the accuracy of the data recording in administrative datasets. No data source recording stillbirths in Australia is 100% accurate and reliable. However, the administrative datasets did perform well. Data linkage is the best way to achieve an adequate sample size for testing predictors of a relatively rare event like stillbirth. Given the opportunity, women are very willing to self-report having had a stillbirth. In fact, over-reporting of stillbirths was more of an issue than under-reporting. In a dataset like the ALWSH, over-reporting can be reduced by looking at the consistency in survey data over time, and removing the obvious errors during data cleaning. The combination of self-reported longitudinal study data and administrative records provides great opportunities for hypothesis testing. However, care should be taken to cross-check and externally validate each dataset wherever possible.
